# HIV-1 protease with leucine zipper fused at N-terminus exhibits enhanced linker amino acid-dependent activity

**DOI:** 10.1186/s12977-018-0413-6

**Published:** 2018-04-14

**Authors:** Fu-Hsien Yu, Chin-Tien Wang

**Affiliations:** Department of Medical Research, Taipei Veterans General Hospital and Institute of Clinical Medicine, National Yang-Ming University School of Medicine, 201, Sec. 2, Shih-Pai Road, Taipei, 11217 Taiwan

**Keywords:** HIV-1, Gag-Pol, P6pol, Protease maturation, Virus maturation, Gag cleavage

## Abstract

**Background:**

HIV-1 protease (PR) activation is triggered by Gag-Pol dimerization. Premature PR activation results in reduced virion yields due to enhanced Gag cleavage. A p6* transframe peptide located directly upstream of protease is believed to play a modulating role in PR activation. Previous reports indicate that the C-terminal p6* tetra-peptide prevents premature PR activation triggered by a leucine zipper (LZ) dimerization motif inserted in the deleted p6* region. To clarify the involvement of C-terminal p6* residues in mitigating enhanced LZ-incurred Gag processing, we engineered constructs containing C-terminal p6* residue substitutions with and without a mutation blocking the p6*/PR cleavage site, and created other Gag or p6* domain-removing constructs. The capabilities of these constructs to mediate virus maturation were assessed by Western blotting and single-cycle infection assays.

**Results:**

p6*-PR cleavage blocking did not significantly reduce the LZ enhancement effect on Gag cleavage when only four amino acid residues were present between the p6* and PR. This suggests that the potent LZ dimerization motif may enhance PR activation by facilitating PR dimer formation, and that PR precursors may trigger sufficient enzymatic activity without breaking off from the PR N-terminus. Enhanced LZ-induced activation of PR embedded in Gag-Pol was found to be independent of the Gag assembly domain. In contrast, the LZ enhancement effect was markedly reduced when six amino acids were present at the p6*-PR junction, in part due to impaired PR maturation by substitution mutations. We also observed that a proline substitution at the P3 position eliminated the ability of p6*-deleted Gag-Pol to mediate virus maturation, thus emphasizing the importance of C-terminal p6* residues to modulating PR activation.

**Conclusions:**

The ability of HIV-1 C-terminal p6* amino acid residues to modulate PR activation contributes, at least in part, to their ability to counteract enhanced Gag cleavage induced by a leucine zipper substituted for a deleted p6*. Changes in C-terminal p6* residues between LZ and PR may affect PR-mediated virus maturation, thus providing a possible method for assessing HIV-1 protease precursor activation in the context of virus assembly.

## Background

The HIV-1 retrovirus contains three major genes (gag, pol and env) and several accessory genes [1]. HIV-1 pol encodes viral enzymes such as protease (PR), reverse transcriptase (RT) and integrase (IN), while gag encodes viral structural proteins. Both Pol and Gag are translated from the same mRNA template. Pol is translated as a Gag-Pol fusion protein associated with a ribosome shift during Gag translation that occurs at a 5% frequency, leading to a Gag-Pol versus Gag expression ratio of approximately 1:20 [2]. Pr160gag-pol and Pr55gag are transported to plasma membranes, where Pr55gag molecules assemble into virus particles [3]; Pr160gag-pol is incorporated into these particles via Pr55gag interaction [4–7]. During or after virus budding, activated PR auto-cleaves from Gag-Pol and mediates virus maturation through the proteolytic processing of Pr55gag and Pr160gag-pol [8]. Pr55gag cleavage yields four main products: matrix (p17; MA), capsid (CA; p24), nucleocapsid (NC; p7) and C-terminal p6 [9]. Two spacer peptides—SP1 (or p2) and SP2 (or p1)—respectively separate NC from CA and p6. Pr160gag-pol cleavage generates RT and IN in addition to the Gag proteins MA, CA and NC. PR-mediated virus maturation is necessary for viral infectivity acquisition [10, 11].

It is generally believed that Gag-Pol dimerization triggers PR activation [3]. In agreement with this assumption, mutations upstream or downstream of PR may significantly reduce PR-mediated Gag cleavage efficiency due to inadequate Gag-Pol dimerization [12–16]. In contrast, the promotion of PR activation as a result of enhanced Gag-Pol dimerization or Gag-PR dimer interaction likely triggers premature or enhanced Pr55gag cleavage, resulting in markedly reduced virus production. [17–19]. Accordingly, preventing premature PR activation is central to virus assembly.

Within Gag-Pol, truncated p1-p6gag is replaced with a transframe region referred to as p6* or p6pol. Located directly adjacent to the PR N-terminus, p6* has been described as playing a role in modulating PR activation even though it lacks a specific structure. Mature HIV-1 PR has a dimeric form, and the removal of p6* from PR precursor is essential for PR to be fully functional [20–23]. Mutations that block p6*-PR cleavage markedly impede PR-mediated virus maturation, implying a suppressive effect of p6* on PR activation [22, 23]. Molecular models suggest that p6* may prevent early PR maturation by inducing instability in the folded PR dimer structure [24–27]. However, virus assembly-associated evidence in support of this assumption is limited, since deletion analysis of p6* function has the potential to compromise virus assembly due to an overlap of p6* with the p6gag budding domain.

To investigate p6* function without affecting the p6gag coding region, we engineered an HIV-1 virus-producing vector by placing the pol coding sequence at the PR-inactivated C-terminus. This construct, designated Dp6*PR, was capable of assembling and processing virus particles in a manner similar to that of wild-type (wt). Replacement of p6* with a leucine-zipper (LZ) dimerization domain has been shown to eliminate virus production as a result of enhanced Gag cleavage, but as few as four C-terminal p6 residues remaining between LZ and PR significantly counteract the LZ enhancement effect [28]. These observations provide supporting evidence that p6* may contribute to the prevention of premature PR activation, but it remains unknown whether a correlation exists between the ability of C-terminal p6* residues to counteract LZ enhancement and their ability to modulate PR maturation. Further, it is unknown whether specific amino acids must be present between LZ and PR in order to counteract the LZ enhancement effect.

To study these questions, we engineered multiple constructs to further analyze the role of C-terminal p6* residues in counteracting LZ enhancement and modulating PR activation. Our results indicate that an HIV-1 PR precursor containing a leucine zipper (LZ) motif linked at the N-terminus eliminated virus particle production associated with enhanced Gag cleavage, suggesting that the HIV-1 PR precursor is capable of exhibiting enhanced enzymatic activity. C-terminal p6* residue substitutions can subvert the Gag cleavage enhancement effect induced by a LZ substitution for p6*, likely the result of interference with PR maturation. While p6*-deleted Gag-Pol (∆p6*fs) containing the last two remaining C-terminal p6* residues was still capable of producing infectious virions following co-expression with Pr55gag, a single amino acid residue change at the deleted p6* region completely removed the ability of ∆p6*fs to mediate virus maturation. Our results confirm the importance of C-terminal p6* residues for the spatiotemporal modulation of PR activity, and provide a virus assembly system for studying HIV-1 protease precursor activation by manipulating linker residues between fused peptides and PR.

## Methods

### Plasmid construction

The parental HIV-1 proviral sequence in this study is HXB2 [29]. The HIV-1 proviral plasmid HIVgpt is considered the backbone of all expression constructs [30]. The constructs used in this research were mostly derived from Dp6*PR, DPR, DWzPR, DWz/PR and DWz//PR. As described previously [31], Dp6*PR contains p6* domain between an inactivated and an active PR. DPR contains BamHI-linked duplicate PR pairs, with the proximal PR was inactivated. DWzPR, DWz/PR and DWz//PR contain leucine zipper replacements of p6* with two, four and six C-terminal p6* residues remaining in the p6*/PR junction, respectively [28]. PSHL and PIDL substitutions for the four C-terminal p6* residues in DWz/PR yielded DWz/PSHL/PR and DWz/PIDL/PR [28]. Primers used for engineering the designated mutations were listed in Table 1.

**Table 1:** Primer sequences used for plasmid construction

Constructs	Forward primer (5′–3′)^a^
DWzPR	5′ CTGTGGATCCT**AACTTC**CCTCAGGTAACGTTATGGCAA 3′-nt 2273
DWz/PR	5′ CGGGATCCT**TCCTTTAACTTC**CCTCAGGTCACGTTATGG 3′-nt 2270
DWz//PR	5′ CGGGATCCT**ACTGTATCCTTTAACTTC**CCTCAGGTCACGTTATGG 3′-nt 2270
DWzPRV/P	5′ CGGGATCCT**AACGTT**CCTCAGATCACGTTATGG 3′-nt 2270
DWz/PRV/P	5′ CGGGATCCT**TCCTTTAACGTT**CCTCAGATCACGTTATGG 3′-nt 2270
DWz//PRV/P	5′ CGGGATCCT**ACTGTATCCTTTAACGTT**CCTCAGATCACGTTATGG 3′-nt 2270
DWz/PSHL/PR	5′ CGGGATCCT**CCCTCTCACCTC**CCTCAGGTCACTCTTTGG 3′-nt 2270
DWz/PIDL/PR	5′ CGGGATCCT**CCCATTGACCTC**CCTCAGGTCACTCTTTGG 3′-nt 2270
DWz/PANF/PR	5′ CGGGATCCT**CCCGCTAACTTC**CCTCAGGTCACTCTTTGG 3′-nt 2270
p6*PSHL	nt 2221-5′ CCGATCGACAAGGAACTGTA**CCCTCTCACCTC**CCTCAG 3′-nt 2258
p6*PIDL	nt 2221-5′ CCGATCGACAAGGAACTGTA**CCCATTGACCTC**CCTCAG 3′-nt 2258
p6*PANF	nt 2221-5′ CCGATCGACAAGGAACTGTA**CCCGCTAACTT**CC 3′-nt 2254

^a^ The numbers at the 3′ and/or 5′ ends denote HIV-1 proviral DNA nucleotide positions. Nucleotides corresponding to mutated amino acid residues are shown in boldface. Most of the primers contain BamHI sites (underlined) to facilitate cloning

V/P, as described previously was created by changing amino acid residues at p6*-PR cleavage site from Phe/Pro into Val/Pro [32]. BamHI-containing forward primers were used to amplify the p6* C-terminal coding fragment containing the desired mutation DWzPRV/P, DWz/PRV/P, DWz//PRV/P or DWz/PANF/PR (Table 1). V/P or HIVgpt served as a template and the reverse primer (nt.3116-90) sequence was 5′-TACATACAAATCATCCATGTTATTGATA-3′. Amplified fragments were digested with BamHI and EcoRV, and subcloned into pBRClaSal/DPR [28]. BamHI-flanking leucine zipper coding fragments derived from PRWzPR [28] were inserted into each pBRClaSal/DPR recombinant, yielding DWzPRV/P, DWz/PRV/P, DWz//PRV/P and DWz/PANF/PR, respectively.

p6*PSHL, p6*PIDL and p6*PANF were constructed by megaprimer PCR method [33] using a forward primer containing the desired mutation (Table 1) and a reverse primer 5′-GGTACAGTCTCAATAGGGCTAATG-3. HIVgpt serves as a template. The amplified fragments were digested with ApaI and BclI, and subcloned into a plasmid cassette pBRCla-Sal that contains HIV-1 coding sequence (from ClaI-nt.831 to SalI-nt.5786). Each mutation-containing pBRCla-Sal cassette was then digested with SpeI and SalI, and ligated into HIVgpt, yielding p6*PSHL, p6*PIDL and p6*PANF.

p6*PSHL, p6*PIDL and p6*PANF were digested with BglII and EcoRV and ligated into DPR digested with BamHI and EcoRV, yielding Dp6*PSHL, Dp6*PIDL/PR and Dp6*PANF/PR, respectively.

GPfs has the Gag and Pol in the same reading frame due to a deletion of the frame shift signal [34]. DWzPR and DWz/PR were digested with BclI. The BclI-flanking fragment containing WzPR and Wz/PR mutations were ligated into PR-inactivated GPfs (fsd) digested with BclI, yielding fsdWzPR and fsdWz/PR. Recombination of fsdWzPR and a Gag-deleted fsd [34] generated construct fsdWzPR∆Gag.

### Cell culture and transfection

293T and HeLa cells were maintained in DMEM supplemented with 10% fetal calf serum. Confluent 293T cells were trypsinized, split 1:10 and seeded onto 10-cm plates 18–24 h before transfection. For each construct, 293T cells were transfected with 20 μg of plasmid DNA by the calcium phosphate precipitation method, with the addition of 50 μM chloroquine to enhance transfection efficiency. Culture media and cells were harvested for protein analysis at 48–72 h post-transfection. When pGAG was co-transfected with the Gag-Pol expression constructs at a DNA ratio of 1:1 or 10 to 1, 10 or 15 μg of pGAG were used with the addition of pBlueScript plasmid DNA to a final quantity of 20 μg DNA. The cells and media were harvested for protein analysis 48–72 h post-transfection.

### Single-cycle infection assays

293T cells were either co-transfected with 10 μg wt or each of the mutant HIVgpt plus 5 μg of the VSV-G protein expression plasmid pHCMV-G [35], or co-transfected with 1 μg of the GPfs or the p6*-deleted GPfs plasmid with 10 μg pGAG plus 5 μg pHCMV-G. At 48 h after transfection, virus-containing supernatants were collected, filtered, diluted, and used to infect HeLa cells. Aliquots of the same filtered supernatants and cell samples were prepared and subjected to Western blot. Adsorption of virions is allowed to proceed in the presence of 4 μg/ml polybrene. Twenty-four hours after infection, cells were trypsinized, split into dishes, and refed with medium containing drug selection cocktail [36]. Selected drug resistant colonies were fixed and stained with 50% methanol containing 0.5% methylene blue. Numbers of drug-resistant colonies were converted into titers (cfu/ml). Infectivity was expressed as the ratio of the mutant titer to the titer of wt, and normalized to Gag protein levels in parallel experiments.

### Western immunoblot analysis

Culture media from transfected 293T cells were filtered through 0.45-µm pore-size and then centrifuged through 2 ml 20% sucrose in TSE (10 mM Tris–HCl, pH 7.5, 100 mM NaCl, 1 mM EDTA) containing 0.1 mM phenylmethylsulfonyl fluoride (PMSF) at 4 °C for 40 min at 274,000 × *g*. Viral pellets and cell lysates mixed with sample buffer were then subjected to SDS-10% PAGE or 4–12% Bis–Tris gradient gels (NuPage Bis–Tris Mini Gels; Thermo Fisher Scientific) followed by immunoblotting analysis as previously described [37]. HIV-1 Gag proteins were probed with an anti-p24gag monoclonal antibody (mouse hybridoma clone 183-H12-5C) from ascites. For HIV-1 RT detection, the primary antibody was rabbit antiserum or a mouse anti-RT monoclonal antibody [38, 39]. Cellular β-actin was detected using a mouse anti-β-actin monoclonal antibody (Sigma). The secondary antibody was either a sheep anti-mouse or a donkey anti-rabbit horseradish peroxidase (HRP)-conjugated antibody (Jackson ImmunoResearch). An enhanced chemiluminescence (ECL) detection system (SuperSignal West Pico Chemiluminescent Substrate; Thermo Fisher Scientific) was used to detect membrane-bound proteins.

### Statistical analysis

Differences between control (wt) and experimental (mutant) groups were assessed using Student’s t-tests. Data are expressed as mean ± standard deviation. Significance was defined as *p < 0.05, **p < 0.01, ***p < 0.001.

## Results

### p6*-PR cleavage blocking does not significantly mitigate leucine zipper-induced Gag cleavage enhancement

**Fig. 1:**
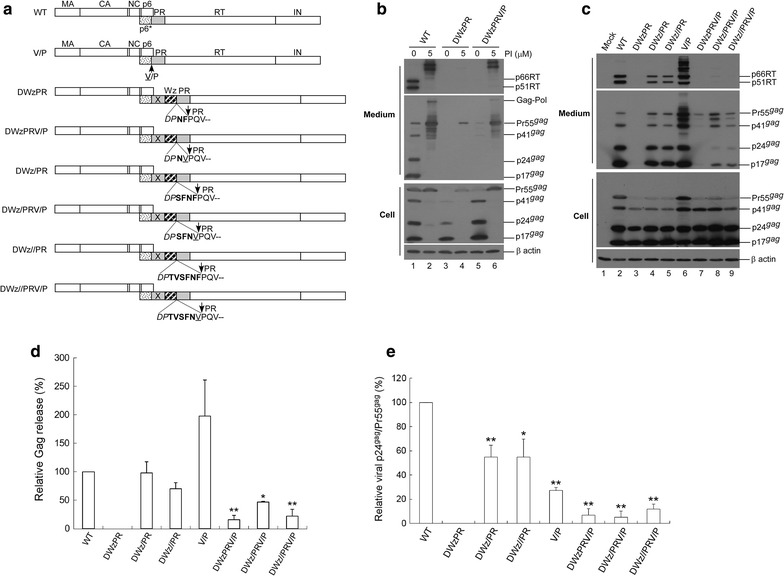
Effects of C-terminal HIV-1 p6* residue substitutions on virus assembly and processing. **a** Schematic representations of HIV-1 Gag and Gag-Pol expression constructs. Indicated are the HIV-1 Gag protein domains MA (matrix), CA (capsid), NC (nucleocapsid), p6, pol-encoded p6*, PR, RT and IN. “X” denotes a PR-inactivated mutation. Arrows indicate PR cleavage sites. Underlined “V” indicates a Val residue substitution for the final C-terminal p6* residue Phe. Striped (Wz) box denotes wild-type (wt) leucine zipper (LZ). Remaining C-terminal p6* residues are in boldface. Altered or additional residues are in italics. **b** Blocking p6*-PR cleavage is insufficient for mitigating the enhanced Gag cleavage incurred by an LZ replacement for a deleted p6* domain. 293T cells were transfected with designated constructs. At 4 h post-transfection, equal amounts of cells were plated on two dishes and either left untreated or treated with saquinavir (a HIV-1 protease inhibitor) at a concentration of 5 μM. Supernatants and cells were collected 48 h post-transfection, prepared, and subjected to Western immunoblotting. **c** Blocking p6*-PR cleavage (V/P mutation) disrupted the function of a C-terminal p6* tetra-peptide for modulating PR activation. 293T cells were transfected with designated constructs. Culture supernatants and cells were collected and subjected to Western immunoblotting at 48–72 h post-transfection. **d** Relative virus assembly efficiencies of HIV-1 mutants. Gag proteins from medium or cell samples were quantified by scanning mutant and wt p24gag-associated band densities from immunoblots. Ratios of total Gag protein levels in medium to those in cells were determined for each construct and compared with wt release levels; release ratios for each mutant were divided by wt ratios in parallel experiments. Error bars indicate standard deviation. *p < 0.05; **p < 0.01. **e** Relative virus particle processing efficiency data for HIV-1 mutants. Virus-associated Pr55gag and p24gag levels were quantified by scanning immunoblot band densities. Ratios of p24gag to p55gag were determined for each mutant and normalized to those of the wt in parallel experiments. Bars indicate standard deviations. *p < 0.05; **p < 0.01

In a previous study we reported that an HIV-1 mutant (DWzPR) containing a LZ dimerization motif adjacent to and upstream of PR was not capable of producing virions due to the strong enhancement of Gag cleavage [28]. DWzPR is derived from Dp6*PR by replacing a deleted p6* with LZ, but retaining the last two C-terminal p6* residues at the LZ/PR junction (Fig. 1a). We observed that this LZ replacement of p6* led to the elimination of virus assembly, likely due to premature PR activation triggered by the LZ. Based on our prior finding that the DWzPR virus assembly defect is PR activity-dependent, and since p6*-PR cleavage is required for fully active PR, we postulated that blocking p6*-PR cleavage within the LZ/PR junction might help restore DWzPR-associated virus production by reducing PR activity. To test this possibility, we substituted Val for the last C-terminal p6* residue (Phe) at the LZ/PR junction of DWzPR and designated the resulting construct as DWzPRV/P (Fig. 1a; note that p6*-PR cleavage site residues were changed from F/P to V/P). Our data indicate that DWzPR virus yields were still hardly detected following the Val substitution unless it was accompanied by treatment with an HIV-1 PR inhibitor (Fig. 1b middle panel, lane 4 vs. lane 5). These results suggest that (a) the blocking of p6*-PR cleavage exerted no major impacts on LZ-induced Gag cleavage enhancement, and (b) a HIV-1 PR precursor containing a LZ motif fused at the N-terminus exhibited enhanced enzymatic activity.

As a control, Val substitution for Phe (referred to as a V/P mutation) significantly reduced virus processing efficiency when tested in a wild-type (wt) HIV-1 Gag/Gag-Pol expression vector (Fig. 1c, middle panel, lanes 2 and 6). Constructs with either four or six remaining C-terminal p6* residues at the LZ/PR junction (DWz/PR or DWz//PR) exhibited particle assembly and processing profiles similar to that of the wt (Fig. 1c middle panel, lanes 4 and 5). While the V/P mutation exerted no major impacts on wt virus production, it significantly reduced DWz/PR and DWz//PR virus yields in addition to impairing virus maturation (Fig. 1c middle panel lanes 4–5 vs. 8–9 and panels d and e). The capacity of the C-terminal p6* tetra-peptide to counteract the LZ enhancement effect and modulate PR activation was subject to weakening by the V/P mutation. This may account, at least in part, for the decreased virus assembly and processing efficiency of DWz/PRV/P and DWz//PRV/P (Fig. 1c middle panel, lanes 8 and 9).

### Specific C-terminal p6* residues are required to modulate PR maturation

**Fig. 2:**
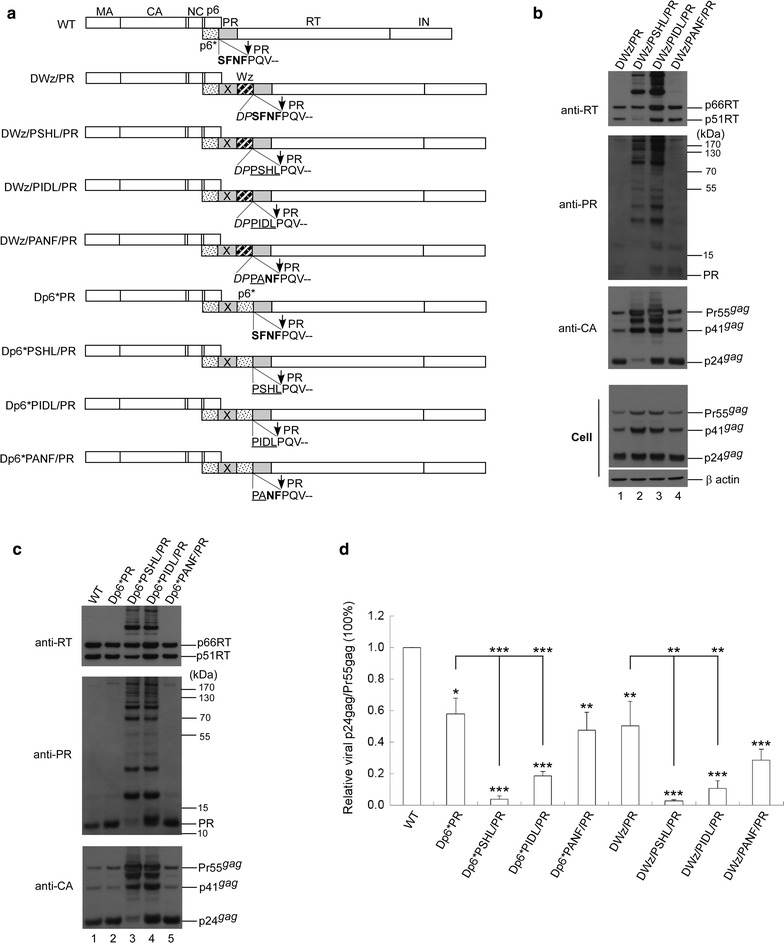
Effects of C-terminal p6* amino acid substitutions on protease maturation and virus processing. **a** Schematic representations of HIV-1 Gag and Gag-Pol expression constructs. HIV-1 Gag protein domains and pol-encoded proteins and the leucine zipper (LZ) motif (striped box, Wz) are indicated as described in the Fig. 1 caption. Also indicated are amino acid residues in the junction area. Two or four C-terminal p6* residues remaining at the LZ/PR junction are shown in boldface. Amino acid changes at the C-terminal p6* tetra-peptide are underlined. Altered or additional residues are in italics. **b**, **c** 293T cells were transfected with designated constructs. Culture supernatants and cells were collected at 48–72 h post-transfection. To detect PR-associated products, aliquots of supernatant samples were separated by 4–12% Bis–Tris gradient gels. Membrane-bound proteins were initially probed with anti-PR serum prior to stripping and probing with anti-RT serum, followed by probing with an anti-p24CA monoclonal antibody. Molecular weight size markers (in kDa) are indicated on right side (middle panels). **d** Relative virus particle processing efficiency of HIV-1 mutants. Virus-associated Pr55gag and p24gag levels were quantified by scanning immunoblot band densities. Ratios of p24gag to p55gag were determined for each mutant and normalized to those of the wt in parallel experiments. Bars indicate standard deviations. *p < 0.05; **p < 0.01; ***p < 0.001

Our results support the proposal that the C-terminal p6* tetra-peptide plays a central role in mitigating PR maturation, likely due to the absence of an intact C-terminal p6* tetra-peptide, which lets LZ dictate the PR maturation process and trigger premature PR activation. To test this hypothesis, we inserted substitution mutations at the C-terminal p6* tetra-peptide without affecting the p6gag amino acid residues. Given our observation of DWzPR exhibiting Gag cleavage enhancement, the last two C-terminal p6* residues (NF) remaining at the LZ/PR junction might be required for PR activation. We therefore engineered a DWz/PANF/PR construct by replacing SF with PA (Fig. 2a). Both the DWz/PSHL/PR and DWz/PIDL/PR constructs contain substitutions for all four C-terminal p6* residues. We found that DWz/PANF/PR displayed a virus assembly and processing profile similar to that of DWz/PR (Fig. 2b, lane 4 vs. lane 1), but evidence from statistical analyses suggest that its virus particle processing was not as efficient as that of DWz/PR (Fig. 2b, d). Whereas DWz/PIDL/PR exhibited readily detected virus-associated p24gag and mature PR, DWz/PSHL/PR had virus-associated Gag or PR mostly present in unprocessed or incompletely processed precursor forms (Fig. 2b, lane 3 vs. lane 2). Similar effects on virus processing were observed when PSHL, PIDL or PANF mutations were cloned into Dp6*PR (Fig. 2c). Western blot data indicate a strong correlation between the virus processing efficiencies of the mutants and their virus-associated mature PR levels. Although Dp6*PANF/PR and DWz/PANF/PR both exhibited relatively low processing efficiency, no statistical significance was noted when compared with their Dp6*PR and DWZ/PR prototypes (Fig. 2d).

**Fig. 3:**
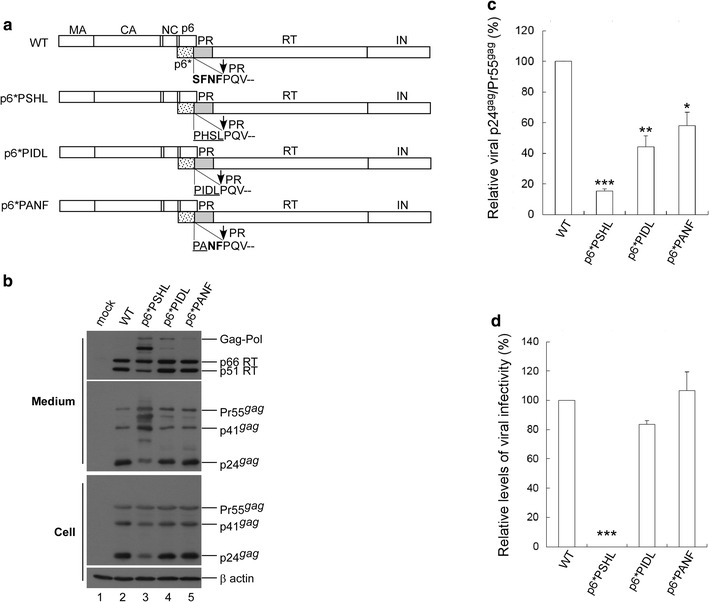
Effects of C-terminal p6* tetra-peptide mutations on virus processing and infectivity. **a** Schematic representations of HIV-1 Gag and Gag-Pol expression constructs. HIV-1 Gag protein domains and pol-encoded proteins are indicated as described in the Fig. 1 caption. Native C-terminal p6* residues are shown in boldface. Altered amino acid residues are underlined. **b** 293T cells were transfected with designated constructs. Culture supernatants and cells were collected 48–72 h post-transfection and subjected to Western immunoblotting. **c** Relative virus particle processing efficiency of HIV-1 mutants. Virus-associated Pr55gag and p24gag levels were quantified by scanning immunoblot band densities. Ratios of p24gag to p55gag were determined for each mutant and normalized to those of the wt in parallel experiments. Bars indicate standard deviations. *p < 0.05; **p < 0.01; ***p < 0.001. **d** Infectivity of HIV-1 mutants. 293T cells were co-transfected with one of the designated constructs plus a VSV-G expression vector. At 48 h post-transfection, supernatants were collected, filtered, and used to infect HeLa cells. Infection and selection of drug-resistant colonies was performed as described in Methods. Infectivity for each mutant was determined as the ratio of mutant titers to wt titers, normalized to Gag protein levels in parallel experiments. ***p < 0.001

To confirm our conclusions, we tested PIDL, PSHL and PANF substitution mutations in a wt HIVgpt backbone (Fig. 3a). Results indicate that neither PIDL nor PANF mutations significantly affected virus infectivity in single cycle infection assays, although both mutations reduced virus processing efficiency (Fig. 3b–d). The data also show that PANF exerted a weaker impact on virus processing and infectivity compared to PIDL. In contrast, the PSHL mutation significantly impaired both virus processing and infectivity (Fig. 3c, d). Combined, these results suggest that specific C-terminal p6* residues, especially the last two, are essential for modulating PR activation.

### A single amino acid residue change eliminated the ability of p6*-deleted Gag-Pol to mediate virus maturation

**Fig. 4:**
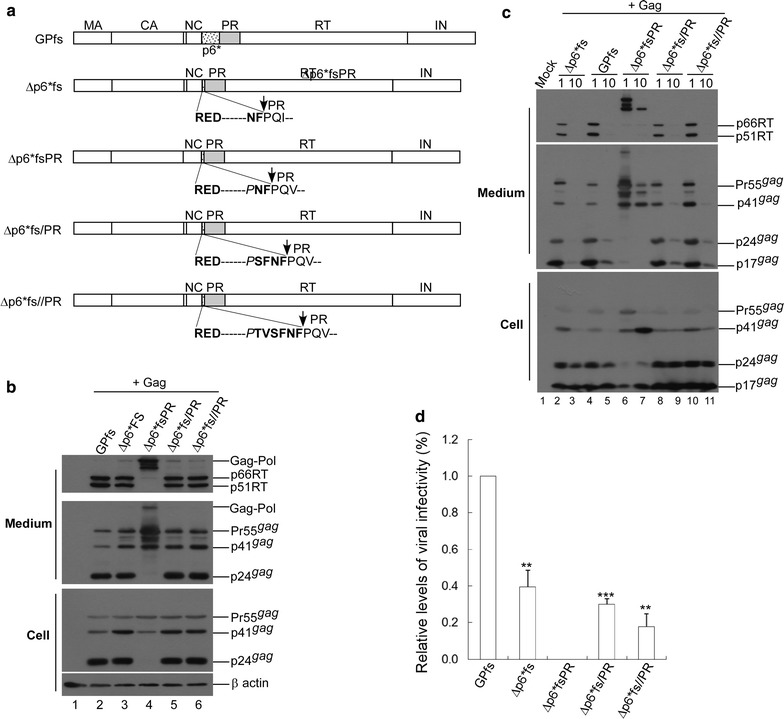
Effects of C-terminal p6* residue substitutions on the capability of p6*-deleted Gag-Pol mutants to mediate virus maturation. **a** Schematic representations of HIV-1 Gag-Pol expression constructs with deletions of most p6* coding sequences. HIV-1 Gag domains, pol-encoded p6*, PR, RT and IN are indicated. All constructs contain a frame shift (fs) mutation forcing gag and pol into the same reading frame. Dashed lines denote deleted p6* regions. Remaining N-terminal and C-terminal p6* residues are indicated in boldface. Altered or foreign residues are in italics. **b**,** c** 293T cells were co-transfected with 10 μg of an HIV-1 Pr55gag expression plasmid (pGAG) and 1 or 10 µg (panel **b**) or 1 µg (panel **c**) of the designated Gag-Pol expression construct. At 48 h post-transfection, cells and supernatants were collected and analyzed by Western immunoblotting. Membrane-bound proteins were initially probed with anti-RT serum, stripped, and probed again with anti-p17MA and anti-p24CA monoclonal antibodies. Indicated are HIV-1 Gag-Pol, 66/51RT, Pr55gag, p41gag, p24gag and p17gag positions. **d** A single amino acid change blocked the capability of p6*-deleted Gag-Pol to confer virus infectivity. 293T cells were co-transfected with 10 µg of an HIV-1 Pr55gag expression vector (pGAG) and 1 µg of one of the designated constructs plus 5 µg of a VSV-G expression vector. At 48 h post-transfection, supernatants were collected, filtered, and used to infect HeLa cells. Infectivity for each Gag-Pol construct was determined as the ratio of mutant titers to wt Gag-Pol titers, normalized to virus-associated p24gag protein levels in parallel experiments. **p < 0.01; ***p < 0.001

The above conclusions agree with our past observations of HIV-1 Gag-Pol (∆p6*fs) with most p6* deleted, but with the final two C-terminal p6* residues still capable of mediating virus maturation, although less efficiently than wt Gag-Pol [40]. To determine if additional changes at C-terminal p6* residues affect the ability of ∆p6*fs to mediate Gag processing, we engineered p6*-deleted constructs with two, four or six C-terminal p6* residues remaining at the p6*/PR junction (respectively designated ∆p6*fsPR, ∆p6*fs/PR and ∆p6*fs//PR). All three contained an additional Pro residue insertion in the deleted p6* region due to cloning procedures (Fig. 4a). All of the p6*-deleted Gag-Pol mutants and wild-type GPfs were co-transfected with a Pr55gag expression vector designated pGAG. Unsurprisingly, virus production was almost completely blocked when each construct was co-expressed with equal amounts of pGAG (10 μg each), presumably due to enhanced Gag cleavage from over-expressed PR activity. The only exception was ∆p6*fsPR, which produced significant amounts of mostly unprocessed or incompletely processed virus-associated Gag (Fig. 4b middle panel, lane 7). All constructs other than ∆p6*fsPR were capable of producing readily detectable virus-associated p24gag and p66/51RT when co-expressed with pGAG at a plasmid DNA ratio of 1:10 (Fig. 4b, c). In contrast, ∆p6*fsPR co-expression with pGAG consistently yielded virions that mostly contained incomplete or unprocessed Gag and RT-associated Gag-Pol (Fig. 4b, lane 4, and Fig. 4c, lane 6). These data suggest that ∆p6*fsPR is profoundly defective in auto-processing and the *in trans* processing of virus particles.

In another test designed to determine whether the p6*-deleted Gag-Pol mutants mediated virus maturation and produced infectious virions, each Gag-Pol construct was co-expressed with pGAG plus a VSV-G envelope expression plasmid. Culture supernatants were collected for protein analysis and used to infect HeLa cells. Our data indicate that with the exception of ∆p6*fsPR, all p6*-deleted Gag-Pol mutants were capable of producing infectious virions with infectivity levels of approximately 20–40% relative to wt GPfs (Fig. 4d). The third N-terminal PR residue at ∆p6*fsPR (Val instead of Ile) apparently does not account for the virus processing defect, since a Val/Ile polymorphism was found at this position. Further, both ∆p6*fs/PR and ∆p6*fs//PR containing a Val polymorphism were capable of mediating virus particle maturation. ∆p6*fsPR contains an inserted proline adjacent to the last two C-terminal p6* residues. Although ∆p6*fs/PR and ∆p6*fs//PR both contain the same proline insertion at the p6*-deleted region, both were still found to be capable of mediating virus maturation. The four remaining C-terminal p6* residues within ∆p6*fs/PR and ∆p6*fs//PR might prevent the Pro insertion from interfering with PR activation.

### Enhanced PR activation due to the LZ replacement of p6* is Gag domain-independent

**Fig. 5:**
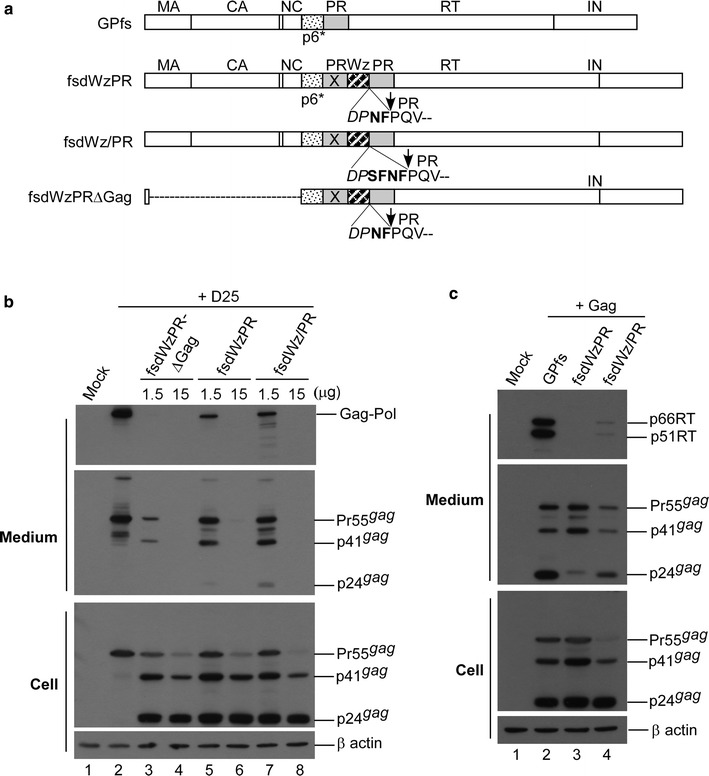
Enhanced Gag-Pol auto-cleavage reduces virus yields and Gag-Pol viral incorporation. **a** Schematic representations of HIV-1 Gag-Pol expression constructs in a gag-pol frame shift (fs) mutation backbone are as described in the Fig. 4 caption. Striped (Wz) box denotes leucine zipper (LZ). Dashed line indicates deleted Gag coding sequence. “X” denotes a PR-inactivated mutation. Remaining C-terminal p6* residues are in boldface. Altered or additional residues are shown in italics. **b**,** c** Leucine zipper-induced Gag-Pol auto-cleavage enhancement leads to reductions in virion yields and Gag-Pol packaging. Indicated amounts of designated plasmids were co-expressed with 15 μg of an HIV-1 protease-defective (D25) Gag/Gag-Pol expression vector (panel b) or co-expressed with pGAG (panel c) at a DNA ratio of 1:10. Culture supernatants and cells were collected at 48–72 h post-transfection and subjected to Western immunoblotting. Indicated are HIV-1 Gag-Pol, 66/51RT, Pr55gag, p41gag and p24gag positions

Given the contribution that Gag makes to PR activation by promoting Gag-Pol dimerization, we hypothesized that Gag removal from DWzPR might impair Gag-Pol dimerization, thereby mitigating the LZ enhancement effect on PR activation and reducing both Gag-Pol auto-cleavage and Gag cleavage efficiency. To test this idea, we engineered GPfs versions of DWzPR with a Gag deletion (fsdWzPR∆Gag) and without one (fsdWzPR) (Fig. 5a). GPfs and a GPfs version of DWz/PR (designated fsdDWz/PR) served as controls. Each construct was co-expressed with D25, a PR-inactivated Gag/Gag-Pol expression plasmid. According to our results, both fsdWzPR and fsdWzPR∆Gag produced barely detectable virus-associated p24gag when co-transfected with D25 at a DNA ratio of 1:10 (Fig. 5b middle panel, lanes 3 and 5). In contrast, virus-associated p24gag was readily detected in fsdWz/PR-plus-D25 co-transfection samples although at a much lower level compared to Pr55gag and p41gag (Fig. 5b, lane 7). Incorporated Gag-Pol deficiency due to premature or enhanced Gag-Pol auto-cleavage might result in insufficient virus processing. The over-expression of fsdWzPR∆Gag and other GPfs mutants led to the complete blocking of virus assembly (Fig. 5b, lanes 4, 6 and 8).

Virus-associated Gag-Pol molecules detected in medium were likely from D25 (Fig. 5b, upper panel). It is possible that D25 Gag-Pol competes with other Gag-Pol mutants in terms of viral incorporation. There is also the possibility that D25 PR-defective Gag-Pol interferes with the ability of incorporated Gag-Pol mutants to mediate virus particle processing. This may partly explain the relatively lower levels of virus-associated p24gag that we observed in fsdWz/PR co-transfection samples (Fig. 5b middle panel lane 7). To study these possibilities, Gag-Pol mutants were co-expressed with pGAG. Results indicate that virus-associated RT and p24gag were both readily detected in wt GPfs or fsdWz/PR co-transfection samples (Fig. 5c). In contrast, virus-associated RT and p24gag were both barely detectable in fsdWzPR co-transfection samples, likely due to a Gag-Pol incorporating defect (Fig. 5c, lane 3).

Combined, these results suggest that an LZ replacement for p6* leads to enhanced Gag-Pol auto-cleavage and associated Gag-Pol incorporation deficiency, and that Gag removal does not reduce the LZ enhancement effect on PR activation.

## Discussion

Even though the removal of p6* from PR precursor is necessary for PR to be fully functional, the enhancement effect of LZ on PR-mediated Gag processing was not significantly compromised by blocking p6*-PR cleavage. Further, we noted that DWz/PRV/P, DWz//PRV/P and other constructs with substitution mutations at the remaining C-terminal p6* tetra-peptide were all capable of producing readily detectable virus-associated Gag, although with processing defects for some of the mutants (Figs. 1, 2). These results suggest that the enhancement of PR activation by LZ may be reduced when as few as six amino acid residues, whether native or foreign, are present between LZ and PR. The hexa-peptide between LZ and PR may serve as a spacer that prevents the potent LZ dimerization motif from facilitating PR dimer formation. Additionally, substitutions at the C-terminal p6* tetra-peptide might interfere with PR maturation, thereby contributing, at least in part, to reduced Gag processing efficiency.

As a result of blocked cleavage at the PR-RT site, HIV-1 PR-RT fusion is capable of mediating virus processing and supporting virus replication [41]. In contrast, p6* appears to be capable of inhibiting PR maturation, and therefore must be removed for PR to be fully active. However, DWzPR virus yields were not significantly restored when p6*-PR cleavage was blocked, suggesting that HIV-1 PR precursors containing a LZ motif fused at the N-terminus may exhibit markedly enhanced enzymatic activity even when free mature PR is not released. Theoretically, PR-associated products with LZ linked at the PR N-terminus may exist in DWzPRV/P transfectant samples due to blocked cleavage at p6*/PR. Since attempts to detect these PR-associated products were unsuccessful, we believe PR may access putative cryptic cleavage sites within the LZ in addition to being self-degrading. Regardless, our findings suggest that PR precursors containing foreign peptides fused at the N-terminus are still capable of being functionally active. Specifically, PR activity may be significantly enhanced when a potent dimerization motif is present at the PR N-terminus.

Although C-terminal p6* substitutions with either PIDL or PANF residues exerted no major impacts on HIVgpt virus infectivity according to single-round infection assays, they did reduce virus processing efficiency (Fig. 3). These data agree with an earlier report that substitutions for C-terminal p6* residues can impair PR maturation, resulting in a virus processing defect [20]. Negative effects of C-terminal p6* tetra-peptide substitution mutations on virus processing become increasingly noticeable when tested with a Dp6*PR backbone (Fig. 2c), presumably due to PR function perturbation by an upstream inactivated PR copy.

According to single-cycle infection assays, ∆p6*fs generated mature infectious virions. In contrast, ∆p6*fsPR (with only one amino acid difference from ∆p6*fs in the linker region) was completely blocked in terms of mediating virus maturation (Fig. 4). ∆p6*fs has a Leu at the P3 position, while ∆p6*fsPR has a foreign Pro insertion at P3. Pro residues were barely detected at the P3 position flanking the PR cleavage site (i.e., the third amino acid residue immediately upstream from the PR substrate cleavage site). Leu was one of several amino acids found at the P3 position [42]. This raises the possibility of P3-Pro disrupting PR maturation, thereby blocking the ability of ∆p6*fsPR to mediate virus processing. Even though they both contain a Pro insertion in the deleted p6* region, ∆p6*fs/PR and ∆p6*fs//PR were still capable of mediating virus maturation, likely due to the containment of an intact C-terminal p6* tetra-peptide SFNF at the p6*/PR junction. This agrees with our observations involving DWz/PR and DWz//PR, both of which contain an intact C-terminal p6* tetra-peptide and are capable of counteracting the LZ enhancement of PR activation.

In addition to containing only four residues between LZ and PR, DWzPR has a Pro at the P3 position, which might contribute to its failure to counteract the LZ enhancement effect on PR-mediated Gag cleavage. C-terminal p6* residues might inhibit PR maturation by destabilizing the structure of the folded PR dimer [43]. The absence of regulated PR dimer structure folding due to mutations at C-terminal p6* residues might allow the potent LZ dimerization motif to facilitate or exert a synergetic effect on Gag-Pol or PR dimer formation via PR dimer stabilization. PR is consequently prematurely activated, resulting in a significant enhancement of Gag processing as was observed for DWzPR and DWzPRV/P.

In conclusion, our findings suggest that an HIV-1 PR precursor containing an N-terminally extended peptide can function efficiently without liberating free mature PR. HIV-1 PR precursor activity might be manipulated via the altering of peptide residues adjacent to the PR N-terminus.
